# The relationship between mental disorders and actual and desired subjective social status

**DOI:** 10.1017/S2045796019000805

**Published:** 2019-12-16

**Authors:** Y. A. de Vries, M. ten Have, R. de Graaf, S. van Dorsselaer, N. M. P. de Ruiter, P. de Jonge

**Affiliations:** 1 Department of Developmental Psychology, University of Groningen, Groningen, The Netherlands; 2 Interdisciplinary Center Psychopathology and Emotion regulation, University Medical Center Groningen, University of Groningen, Groningen, The Netherlands; 3 Netherlands Institute of Mental Health and Addiction, Utrecht, The Netherlands; 4 University College Groningen, University of Groningen, Groningen, The Netherlands

**Keywords:** Mental disorders, remission, social status, subjective social status

## Abstract

**Aims:**

Mental disorders are associated with lower subjective social status (SSS), but a more nuanced understanding of this relationship is needed. We examined the influence of disorder age of onset and recency on SSS and studied whether mental disorders are also associated with the discrepancy between *actual* and *desired* SSS.

**Method:**

Data are from the baseline and second wave of the Netherlands Mental Health Survey and Incidence Study-2 (NEMESIS-2). Mental disorders were assessed with the Composite International Diagnostic Interview (CIDI 3.0), while both actual and desired SSS were assessed with a ten-rung ladder. Linear regression was used to examine the association between mental disorders and SSS.

**Results:**

Of 5303 participants, 2237 had a lifetime mental disorder at baseline. These participants reported significantly lower actual SSS (6.28) at follow-up than healthy participants (6.66, *B* = −0.38 [95% CI −0.48 to −0.27], *p* < 0.001) and a significantly greater actual-desired SSS discrepancy (1.14 *v*. 1.05 after controlling for actual SSS, *B* = 0.09 [0.01–0.17], *p* = 0.024). Lower age of onset of the first mental disorder was marginally significantly associated with lower actual SSS (*B* = 0.006 [0.000–0.012], *p* = 0.046). More recent disorders were also associated with lower actual SSS (*B* = 0.015 [0.005–0.026], *p* = 0.005), such that participants whose disorder remitted ⩾6 years before baseline were statistically indistinguishable from healthy participants.

**Conclusions:**

Lifetime mental disorders are associated with lower actual SSS and a slightly greater discrepancy between actual and desired SSS. However, people with mental disorders in (long-term) remission have a similar social status as healthy participants.

## Introduction

Mental disorders are associated with lower socioeconomic status (SES) (Lorant *et al*., 2003; Hudson, 2005). They are, for instance, associated with premature termination of education (Breslau *et al*., 2008; Lee *et al*., 2009) and reduced earnings (Kessler *et al*., 2008; Levinson *et al*., 2010). Causality appears to run in both directions: low SES increases the risk of mental disorders, while the presence of mental disorders also increases the risk of low SES (Johnson *et al*., 1999; Elovainio *et al*., 2012; Pino *et al*., 2018). While SES has traditionally been indicated by objective measures such as education, occupational status and income, more recently interest has shifted to examining subjective social status (SSS), a person's subjective judgement of their social position (Adler and Epel, 2000). It is thought that SSS may represent a kind of ‘cognitive averaging’ of various SES indicators (Singh-Manoux *et al*., 2003) and hence might be a more comprehensive measure than traditional SES indicators. SSS has generally been found to be associated with (mental) health outcomes even after controlling for objective SES (Adler and Epel, 2000; Singh-Manoux *et al*., 2003, 2005; Operario *et al*., 2004; Hu *et al*., 2005; Franzini and Fernandez-Esquer, 2006; Adler *et al*., 2008; Collins and Goldman, 2008; Demakakos *et al*., 2008; Hamad *et al*., 2008; Leu *et al*., 2008; Wong *et al*., 2008; Sakurai *et al*., 2010; Wolff *et al*., 2010; Karvonen and Rahkonen, 2011; McLaughlin *et al*., 2012; Miyakawa *et al*., 2012; Subramanyam *et al*., 2012; Euteneuer, 2014; Honjo *et al*., 2014; Quon and McGrath, 2014; Scott *et al*., 2014; Präg *et al*., 2016; Hoebel *et al*., 2017; Chen *et al*., 2019), which suggests that SSS is indeed a more comprehensive measure of SES or that a person's subjective sense of social status matters over and above objective SES.

However, research to date is limited in a number of respects. First, previous studies have relied upon symptom questionnaires rather than examining diagnosable mental disorders, with only a few exceptions (McLaughlin *et al*., 2012; Honjo *et al*., 2014; Scott *et al*., 2014; Chen *et al*., 2019). While symptom questionnaires are useful as screening tools, they are ‘context free’ and hence cannot distinguish between mental disorders and normal distress, and tend to result in large numbers of false positives (Henkel *et al*., 2003; Vilagut *et al*., 2016). Second, it is important to better understand other variables that affect this relationship to provide starting points for ameliorating the SSS of people with mental disorders, for instance by focusing on particular high-risk groups. In this study, we focus on disorder age of onset and remission. Given the effects of early-onset mental disorders on educational attainment (Breslau *et al*., 2008; Lee *et al*., 2009), early-onset disorders might have particularly large associations with SSS as well. It also seems plausible that remission of mental disorders is associated with improvement in SSS, but to date, it is unknown whether participants in long-term remission from mental disorders still have a lower SSS than participants who never suffered from a mental disorder. Third, to our knowledge, no study has examined the association of mental disorders with the discrepancy between *actual* and *desired* SSS. Previous research has, however, examined the effect of a counterfactual SSS by asking single mothers and unemployed persons what their social status would have been if they had not become single parents or unemployed (Euteneuer *et al*., 2019). This study found that the discrepancy between a person's actual and their counterfactual SSS significantly predicted symptoms of stress and depression, even after controlling for actual SSS. This suggests that desired SSS might also be related to mental health, over and above the associations with actual SSS.

In the current study, we aimed to shed more light on the relationship between mental disorders and SSS by examining the role of disorder age of onset and remission, and by also considering the role of the discrepancy between actual and desired SSS.

## Methods

### Participants

We used data from the first two waves of the Netherlands Mental Health Survey and Incidence Study-2 (NEMESIS-2). NEMESIS-2 is a psychiatric epidemiological cohort study in a representative sample of the adult population of the Netherlands. Participants were selected by means of a multistage, stratified sampling procedure, with one respondent (aged 18–64) being randomly sampled from randomly selected households from randomly selected municipalities. Face-to-face interviews were performed with each respondent. In the first wave (T0), which took place between November 2007 and July 2009, 6.646 individuals participated (65.1% response rate). The sample was nationally representative, with the exception that younger individuals were somewhat under-represented (de Graaf *et al*., 2010). All T0 respondents were approached for participation in a second wave (T1) 3 years later, from November 2010 to June 2012. A total of 5.303 participants were interviewed again (80.4% response rate among non-deceased participants). T1 non-respondents were younger, lower educated and more frequently unemployed than T1 respondents, but there was no significant association between 12-month mental disorders at T0 and attrition (de Graaf *et al*., 2013).

All procedures involving human subjects were approved by the Medical Ethics Review Committee for Institutions on Mental Health Care. Written informed consent was obtained from all respondents. Further details about the study design are provided elsewhere (de Graaf *et al*., 2010).

### Measures

Lifetime DSM-IV diagnoses for mental disorders were assessed at T0 by means of the Composite International Diagnostic Interview (CIDI) version 3.0, a fully-structured diagnostic interview administered by trained lay interviewers (Kessler and Üstün, 2004). The disorders assessed included mood and anxiety disorders (major depressive disorder, dysthymia, bipolar disorder, generalised anxiety disorder, panic disorder with or without agoraphobia, agoraphobia without panic disorder, specific phobia and social phobia), impulse control disorders (attention-deficit/hyperactivity disorder [ADHD], conduct disorder and oppositional defiant disorder) and substance use disorders (alcohol or drug abuse and dependence). Due to concerns about recall bias, impulse control disorders were only assessed in respondents aged 45 and below. CIDI diagnoses generally have good validity compared to clinical reappraisal interviews (Haro *et al*., 2006). The CIDI was also used to assess the age of onset, using a series of recall probes that have been shown to yield more plausible distributions of age of onset than conventional recall questions (Knäuper *et al*., 1999). In our analyses, we used age of onset as a continuous variable to test its association with SSS and also categorised it into four categories (4–12, 13–19, 20–29, 30–64) to further examine the association between early- or late-onset mental disorders and SSS. Because very few participants had an onset of substance use disorder before the age of 13, we categorised age of onset into three categories (4–19, 20–29, 30–64) for substance use disorders. Recency of each mental disorder was assessed by asking respondents whether they experienced symptoms in the past 12 months and, if not, at what age they last experienced symptoms. Like age of onset, we used recency as a continuous variable to test its association with SSS and also categorised it into four categories (<1 year before T0, 1–5, 6–10, >10 years) to further examine the association between recent or long-remitted mental disorders and SSS.

SSS was assessed at T1 using the MacArthur subjective social status scale, the most widely used scale for SSS (Adler and Epel, 2000). Respondents were presented with a picture of a ten-rung ladder, described as: ‘Think of this ladder as representing where people stand in the Netherlands. At the top of the ladder are the people who are the best off – those who have the most money, the most education and the most respected jobs. At the bottom are the people who are the worst off – who have the least money, least education, and the least respected jobs or no job. The higher up you are on the ladder, the closer you are to the people at the very top; the lower you are, the closer you are to the people at the very bottom.’ They were then asked to place an X on the rung where they thought they stood at this time in their life (*actual SSS*). In another picture of a ten-rung ladder, they were asked to place an X on the rung where they would like to stand (*desired SSS*). We calculated a difference by subtracting the actual SSS from the desired SSS (*actual*–*desired SSS discrepancy*). We use *SSS* as an umbrella term for these concepts.

As objective SES indicators, we used education (primary education or lower secondary education, higher secondary education, higher professional education or university), paid employment situation (employed *v*. not employed), household income category (low, middle or high) and living situation (with partner *v*. not with partner). All objective SES indicators were assessed at T0.

Missingness was very limited (<1%) for all variables except income (9.65% unweighted missingness). To retain participants with missing data for income in our analyses, we included a ‘missing answer’ category in our categorical income variable.

### Analyses

We used linear regression to assess the association of specific lifetime mental disorders at baseline with actual SSS and the discrepancy between actual and desired SSS at follow-up. For subsequent analyses examining the relationship of age of onset and recency to actual SSS and the actual–desired SSS discrepancy, we examined any disorder and the following disorder categories: mood or anxiety disorders, impulse control disorders and substance use disorders. For age of onset, we used dummy variables to compare respondents with a disorder onset in a given age category to respondents without a disorder (group). Hence, this analysis tests whether each age of onset group is significantly associated with SSS compared to participants without a disorder (group). Within the group of participants with a disorder, we also tested the association of age of onset (as a continuous variable) with actual SSS and the actual–desired SSS discrepancy. In contrast to the analysis with dummy variables, this analysis tests whether certain ages of onset are more strongly associated with SSS than other ages of onset, *given the presence of a disorder*. Because impulse control disorders had an early age of onset (<20 years of age) by definition, we did not include tests for age of onset for this disorder category. We performed analogous analyses to examine mental disorder recency.

All analyses were performed twice: the first model only controlled for age and gender; a second model also controlled for objective SES. In models for the actual–desired SSS discrepancy, we additionally controlled for actual SSS in both models, as actual SSS and the actual–desired SSS discrepancy are related (i.e. lower actual SSS would result in a larger discrepancy, all other things being equal). All analyses were performed in Stata, using survey commands to account for the clustering and weighting due to the complex sampling design.

## Results

### Baseline demographics

Demographic characteristics of the sample by the presence or absence of lifetime disorders and by age of onset of lifetime mental disorder are presented in Table 1. There were no significant differences between the groups with regard to gender and educational achievement. However, participants with the first onset of a disorder prior to age 20 were significantly younger at the time of the interview (37.2 years) than healthy participants (42.5 years) and participants with the first onset of a disorder at age 20 or later (46.4 years). Participants with both early- and late-onset disorders were significantly more likely to be unemployed or on disability leave (9.7 and 11.2%, respectively) than healthy participants (3.8%). While participants with late-onset disorders and healthy participants were about as likely to have a low income (20.5 and 21.7%, respectively), participants with an early-onset disorder were significantly more likely to have a low income (35.2%). Participants with an early-onset disorder were also less likely to live with a partner (56.9%) than participants with a late-onset disorder (69.3%) or healthy participants (71.9%).

**Table 1.** Baseline characteristics of participants with or without lifetime mental disorders in NEMESIS-2

		First onset of a disorder		
	No disorder (*N* = 3066)	<20 years of age (*N* = 1290)	⩾20 years of age (*N* = 946)	*p*-value
Age, mean	42.5	37.2	46.4	<0.001
Sex (% female)	49.6	48.5	50.9	0.69
Education (%)				
Primary/lower secondary	29.0	31.4	27.8	
Higher secondary	40.7	43.2	42.5	
Higher professional/university	30.3	25.3	29.7	0.11
Employment (%)				
Employed	72.7	67.9	68.9	
Homemaker	11.4	9.9	11.0	
Student	6.5	10.1	2.0	
Unemployed/disability	3.8	9.7	11.2	
Retired/other	5.6	2.3	6.9	<0.001
Living situation (%)				
With partner	71.9	56.9	69.3	
Single parent	4.4	5.4	7.8	
Single (without children)	12.4	19.1	18.1	
With others	11.4	18.5	4.8	<0.001
Income (%)				
Low	20.5	35.2	21.7	
Middle	40.9	35.9	44.5	
High	25.8	18.8	23.5	
No answer	12.8	10.1	10.3	<0.001

### Lifetime mental disorders and SSS

Most disorders were associated with a statistically significantly lower actual SSS (Table 2, model 1), with the exception (*p* = 0.052–0.127) of agoraphobia (without panic), panic disorder, ADHD and drug abuse. Among the mood disorders, dysthymia and bipolar disorder were associated with a much lower actual SSS (−0.96 and −0.99, respectively) than major depression (−0.40), while among the substance use disorders, alcohol or drug dependence was associated with a much lower actual SSS (−0.67 and −1.04) than abuse (−0.28 and −0.30). Participants with any lifetime mental disorder had a mean actual SSS of 6.28, which was 0.38 (95% CI 0.27–0.48, *p* < 0.001) lower than the mean actual SSS of participants without a lifetime mental disorder. Controlling for objective SES attenuated the magnitude of associations (e.g. from −0.38 to −0.26 for any disorder, Table 2, model 2). All disorder groups remained significantly associated with lower actual SSS, although statistical significance was lost for some individual disorders.

**Table 2.** Effect of lifetime mental disorders on actual SSS and on the discrepancy between actual and desired SSS

		Actual SSS			Discrepancy		
			Model 1	Model 2		Model 1	Model 2
Disorder	*n*	Mean	*B* [95% CI]	*B* [95% CI]	Mean	*B* [95% CI]	*B* [95% CI]
Agoraphobia without panic	48	5.96	−0.54 [−1.08 to 0.00]	−0.35 [−0.76 to 0.06]	1.15	0.06 [−0.26 to 0.38]	0.06 [−0.24 to 0.36]
Generalised anxiety disorder	252	6.10	−0.42 [−0.79 to −0.04]*	−0.24 [−0.57 to 0.09]	1.17	0.08 [−0.07 to 0.23]	0.07 [−0.08 to 0.23]
Panic disorder	198	6.25	−0.25 [−0.53 to 0.03]	−0.19 [−0.44 to 0.05]	1.13	0.04 [−0.21 to 0.29]	0.04 [−0.21 to 0.30]
Social phobia	516	6.13	−0.40 [−0.60 to −0.21]***	−0.28 [−0.46 to −0.09]**	1.19	0.11 [−0.06 to 0.27]	0.10 [−0.06 to 0.26]
Specific phobia	439	6.19	−0.33 [−0.56 to −0.09]**	−0.17 [−0.39 to 0.06]	1.09	0.00 [−0.16 to 0.16]	−0.00 [−0.16 to 0.15]
Any anxiety disorder	1080	6.21	−0.36 [−0.50 to −0.22]***	−0.21 [−0.34 to −0.08]**	1.13	0.05 [−0.07 to 0.17]	0.04 [−0.07 to 0.15]
Major depression	1014	6.17	−0.40 [−0.56 to −0.24]***	−0.30 [−0.45 to −0.14]***	1.19	0.13 [0.02 to 0.24]*	0.12 [0.01 to 0.24]*
Dysthymia	80	5.55	−0.96 [−1.51 to −0.41]***	−0.66 [−1.16 to −0.17]**	0.95	−0.14 [−0.70 to 0.43]	−0.21 [−0.78 to 0.35]
Bipolar disorder	69	5.52	−0.99 [−1.55 to −0.44]***	−0.54 [−1.08 to 0.00]	1.56	0.48 [−0.16 to 1.11]	0.45 [−0.16 to 1.06]
Any mood disorder	1091	6.14	−0.45 [−0.61 to −0.30]***	−0.32 [−0.47 to −0.17]***	1.22	0.16 [0.05 to 0.27]**	0.15 [0.04 to 0.26]**
Attention-deficit/hyperactivity disorder	71	5.94	−0.56 [−1.29 to 0.16]	−0.12 [−0.82 to 0.58]	1.10	0.01 [−0.43 to 0.45]	−0.03 [−0.45 to 0.40]
Conduct disorder	167	5.95	−0.56 [−1.04 to −0.09]*	−0.33 [−0.76 to 0.09]	1.55	0.48 [0.08 to 0.88]*	0.46 [0.08 to 0.83]*
Oppositional-defiant disorder	77	5.82	−0.69 [−1.26 to −0.11]*	−0.57 [−1.10 to −0.03]*	1.18	0.09 [−0.20 to 0.38]	0.08 [−0.21 to 0.38]
Any impulse control disorder	266	5.88	−0.65 [−0.99 to −0.30]***	−0.41 [−0.72 to −0.10]*	1.33	0.25 [−0.03 to 0.53]	0.22 [−0.05 to 0.49]
Alcohol abuse	765	6.25	−0.28 [−0.45 to −0.11]**	−0.24 [−0.40 to −0.09]**	1.14	0.06 [−0.09 to 0.21]	0.05 [−0.09 to 0.20]
Alcohol dependence	114	5.84	−0.67 [−1.13 to −0.21]**	−0.18 [−0.64 to 0.29]	1.41	0.32 [−0.03 to 0.68]	0.27 [−0.06 to 0.59]
Drug abuse	202	6.20	−0.30 [−0.67 to 0.06]	−0.07 [−0.38 to 0.23]	1.17	0.08 [−0.15 to 0.31]	0.06 [−0.16 to 0.28]
Drug dependence	111	5.48	−1.04 [−1.62 to −0.45]***	−0.71 [−1.25 to −0.17]*	1.65	0.57 [0.04 to 1.10]*	0.53 [0.02 to 1.04]*
Any substance use disorder	1017	6.19	−0.38 [−0.52 to −0.24]***	−0.25 [−0.37 to −0.13]***	1.17	0.10 [−0.04 to 0.25]	0.09 [−0.05 to 0.22]
Any disorder	2302	6.28	−0.38 [−0.48 to −0.27]***	−0.26 [−0.36 to −0.17]***	1.14	0.09 [0.01 to 0.17]*	0.08 [0.00 to 0.16]*

*Notes*: Model 1 only controls for age and gender. Model 2 additionally controls for education, income, job status and living situation. For the discrepancy, both models additionally control for actual SSS. The reference group consists of participants without that particular disorder (group).

*p < 0.05, **p < 0.01, ***p < 0.001.

There were few associations between specific mental disorders and the actual–desired SSS discrepancy (controlling for actual SSS, Table 2). However, participants with any lifetime mental disorder had a mean actual–desired SSS discrepancy of 1.14, which was 0.09 (95% CI 0.01–0.17) larger than that of participants without a lifetime mental disorder. Major depression and any mood disorder were also associated with small increases in the discrepancy (*B* = 0.13 and 0.16), while conduct disorder and drug dependence were associated with relatively large increases in the actual–desired SSS discrepancy (*B* = 0.48 and 0.57). Associations were essentially unchanged after controlling for objective SES.

### Age of onset, recency and SSS

Tables 3 and 4 show the association between mental disorders and SSS, separated out by disorder category and by age of onset (Table 3) or recency (Table 4) category. Having a lifetime mental disorder was significantly associated with actual SSS for each age of onset category and each disorder category (regression coefficients ranging from −1.01 to −0.25, *p* < 0.001–0.015). Associations were somewhat attenuated when controlling for objective SES, but most remained significant (Table 3, model 2). There were few associations between any age of onset and disorder category and the actual–desired SSS discrepancy. Only having any lifetime mental disorder with late onset (between age 30 and 64) was associated with the actual–desired SSS discrepancy (*B* = 0.13, *p* = 0.021); this association remained unchanged after controlling for objective SES.

**Table 3.** Effect of mental disorders on actual SSS and the SSS discrepancy, by age of onset category

Age at onset	Actual SSS				Discrepancy			
	Model 1		Model 2		Model 1		Model 2	
	*B* [95% CI]	*p*-value	*B* [95% CI]	*p*-value	*B* [95% CI]	*p*-value	*B* [95% CI]	*p*-value
Any disorder								
4–12	−0.52 [−0.69 to −0.35]***	<0.001	−0.36 [−0.51 to −0.21]***	<0.001	0.06 [−0.07 to 0.19]	0.376	0.05 [−0.08 to 0.17]	0.478
13–19	−0.37 [−0.58 to −0.16]***	<0.001	−0.25 [−0.45 to −0.04]*	0.019	0.12 [−0.03 to 0.27]	0.122	0.11 [−0.04 to 0.27]	0.159
20–29	−0.27 [−0.43 to −0.11]***	<0.001	−0.30 [−0.44 to −0.16]***	<0.001	0.06 [−0.07 to 0.19]	0.389	0.06 [−0.08 to 0.19]	0.410
30–64	−0.25 [−0.41 to −0.09]**	0.002	−0.09 [−0.23 to 0.06]	0.231	0.13 [0.02 to 0.24]*	0.021	0.13 [0.02 to 0.24]*	0.024
No disorder	0.00		0.00		0.00			
Mood or anxiety								
4–12	−0.53 [−0.72 to −0.34]***	<0.001	−0.39 [−0.56 to −0.21]***	<0.001	0.10 [−0.04 to 0.25]	0.168	0.09 [−0.05 to 0.23]	0.201
13–19	−0.47 [−0.77 to −0.18]**	0.002	−0.28 [−0.57 to 0.00]	0.051	0.08 [−0.15 to 0.32]	0.486	0.07 [−0.17 to 0.31]	0.564
20–29	−0.40 [−0.72 to −0.08]*	0.015	−0.41 [−0.70 to −0.13]**	0.005	0.23 [−0.01 to 0.47]	0.062	0.23 [−0.01 to 0.47]	0.056
30–64	−0.24 [−0.40 to −0.08]**	0.004	−0.11 [−0.26 to 0.04]	0.152	0.09 [−0.02 to 0.21]	0.113	0.10 [−0.01 to 0.22]	0.086
No disorder	0.00		0.00		0.00			
Impulse control								
4–12	−0.50 [−0.86 to −0.15]**	0.005	−0.24 [−0.56 to 0.08]	0.140	0.13 [−0.16 to 0.42]	0.378	0.10 [−0.17 to 0.38]	0.462
13–19	−1.01 [−1.82 to −0.20]*	0.015	−0.83 [−1.55 to −0.12]*	0.022	0.55 [−0.11 to 1.22]	0.099	0.52 [−0.10 to 1.15]	0.100
No disorder	0.00		0.00		0.00			
Substance use								
4–19	−0.33 [−0.57 to −0.10]**	0.005	−0.18 [−0.38 to 0.02]	0.070	0.19 [−0.01 to 0.38]	0.058	0.18 [−0.01 to 0.36]	0.066
20–29	−0.32 [−0.52 to −0.13]**	0.001	−0.32 [−0.48 to −0.17]***	<0.001	−0.04 [−0.22 to 0.13]	0.627	−0.05 [−0.23 to 0.12]	0.553
30–64	−0.64 [−0.88 to −0.40]***	<0.001	−0.32 [−0.53 to −0.11]**	0.003	0.10 [−0.17 to 0.37]	0.471	0.05 [−0.21 to 0.31]	0.700
No disorder	0.00		0.00		0.00		0.00	

*Notes*: Model 1 only controls for age and gender. Model 2 additionally controls for education, income, job status and living situation. For the discrepancy, both models additionally control for actual SSS. The reference group consists of participants without that particular disorder (group).

*p < 0.05, **p < 0.01, ***p < 0.001.

**Table 4.** Effect of mental disorders on actual SSS and the SSS discrepancy, by recency category

Recency	Actual SSS				Discrepancy			
	Model 1		Model 2		Model 1		Model 2	
	*B* [95% CI]	*p*-value	*B* [95% CI]	*p*-value	*B* [95% CI]	*p*-value	*B* [95% CI]	*p*-value
Any disorder								
Past year	−0.62 [−0.77 to −0.47]***	<0.001	−0.36 [−0.50 to −0.22]***	<0.001	0.11 [−0.02 to 0.23]	0.103	0.08 [−0.04 to 0.20]	0.183
1–5 years	−0.40 [−0.60 to −0.20]***	<0.001	−0.35 [−0.53 to −0.17]***	<0.001	0.13 [−0.02 to 0.28]	0.091	0.13 [−0.02 to 0.28]	0.089
6–10 years	−0.09 [−0.26 to 0.07]	0.255	−0.14 [−0.26 to −0.01]*	0.037	0.03 [−0.10 to 0.16]	0.649	0.04 [−0.09 to 0.16]	0.552
>10 years	−0.12 [−0.31 to 0.06]	0.198	−0.14 [−0.32 to 0.04]	0.12	0.06 [−0.09 to 0.21]	0.445	0.07 [−0.08 to 0.22]	0.368
No disorder	0.00		0.00					
Mood or anxiety								
Past year	−0.63 [−0.80 to −0.46]***	<0.001	−0.40 [−0.56 to −0.25]***	<0.001	0.17** [0.04 to 0.29]	0.008	0.15* [0.03 to 0.26]	0.011
1–5 years	−0.43 [−0.67 to −0.19]***	<0.001	−0.36 [−0.56 to −0.16]***	<0.001	0.06 [−0.12 to 0.24]	0.525	0.06 [−0.12 to 0.24]	0.482
6–10 years	−0.15 [−0.31 to 0.01]	0.071	−0.16 [−0.30 to −0.01]*	0.035	0.02 [−0.14 to 0.18]	0.799	0.03 [−0.12 to 0.18]	0.712
>10 years	−0.2 [−0.52 to 0.12]	0.225	−0.2 [−0.49 to 0.09]	0.184	0.18 [−0.06 to 0.43]	0.144	0.19 [−0.06 to 0.44]	0.133
No disorder	0.00		0.00					
Impulse control								
Past year	−0.65 [−1.29 to −0.00]*	0.049	−0.07 [−0.66 to 0.53]	0.827	0.18 [−0.26 to 0.62]	0.423	0.11 [−0.32 to 0.53]	0.615
1–5 years	−1.48 [−2.53 to −0.43]**	0.006	−1.07 [−2.02 to −0.12]*	0.028	1.26* [0.18 to 2.34]	0.022	1.22* [0.20 to 2.23]	0.019
6–10 years	−0.73 [−1.38 to −0.09]*	0.025	−0.68 [−1.22 to −0.13]*	0.015	0.08 [−0.33 to 0.48]	0.711	0.07 [−0.31 to 0.44]	0.723
>10 years	−0.17 [−0.62 to 0.29]	0.473	−0.27 [−0.62 to 0.07]	0.114	−0.14 [−0.39 to 0.12]	0.294	−0.12 [−0.37 to 0.14]	0.361
No disorder	0.00		0.00					
Substance use								
Past year	−0.74 [−1.01 to −0.46]***	<0.001	−0.42 [−0.68 to −0.15]**	0.002	0.10 [−0.18 to 0.38]	0.478	0.05 [−0.21 to 0.32]	0.698
1–5 years	−0.39 [−0.73 to −0.05]*	0.023	−0.25 [−0.56 to 0.06]	0.111	0.24 [−0.01 to 0.50]	0.061	0.22 [−0.02 to 0.47]	0.075
6–10 years	−0.14 [−0.45 to 0.16]	0.349	−0.19 [−0.41 to 0.02]	0.081	0.09 [−0.13 to 0.31]	0.420	0.10 [−0.12 to 0.32]	0.362
>10 years	−0.18 [−0.42 to 0.06]	0.146	−0.14 [−0.35 to 0.08]	0.21	0.02 [−0.19 to 0.24]	0.842	0.02 [−0.19 to 0.23]	0.850
No disorder	0.00		0.00					

*Notes*: Model 1 only controls for age and gender. Model 2 additionally controls for education, income, job status and living situation. For the discrepancy, both models additionally control for actual SSS. The reference group consists of participants without that particular disorder (group).

*p < 0.05, **p < 0.01, ***p < 0.001.

Age of onset as a continuous variable was marginally significantly associated with actual SSS among those with any disorder (*B* = 0.006, *p* = 0.046) and among those with substance use disorder specifically (*B* = −0.016, *p* = 0.041), but not with the discrepancy between actual and desired SSS (*B* = −0.007 to 0.002, *p* = 0.300–563) (see Table 5, model 1). Younger age of onset tended to be associated with a lower actual SSS than later age of onset among those with any disorder, while the pattern was remarkably reversed for substance use disorder. After controlling for objective SES indicators, age of onset was no longer significantly related to actual SSS (Table 5, model 2).

**Table 5.** Association of age of onset and recency with actual SSS and the SSS discrepancy, in participants with any disorder or a specific disorder category

	Actual SSS				Discrepancy			
	Model 1		Model 2		Model 1		Model 2	
	*B* [95% CI]	*p*-value	*B* [95% CI]	*p*-value	*B* [95% CI]	*p*-value	*B* [95% CI]	*p*-value
Age of onset								
Any disorder	0.006 [0.000 to 0.012]*	0.046	0.006 [−0.000 to 0.012]	0.052	0.002 [−0.003 to 0.007]	0.380	0.003 [−0.002 to 0.008]	0.279
Mood or anxiety	0.004 [−0.003 to 0.011]	0.240	0.004 [−0.004 to 0.011]	0.345	0.002 [−0.004 to 0.007]	0.563	0.003 [−0.003 to 0.008]	0.368
Substance use	−0.016 [−0.032 to −0.001]*	0.041	−0.006 [−0.020 to 0.008]	0.379	−0.007 [−0.019 to 0.006]	0.300	−0.008 [−0.019 to 0.003]	0.143
Recency								
Any disorder	0.015 [0.005 to 0.026]**	0.005	0.005 [−0.005 to 0.014]	0.358	−0.003 [−0.009 to 0.004]	0.452	−0.001 [−0.007 to 0.006]	0.827
Mood or anxiety	0.016 [0.000 to 0.032]*	0.046	0.004 [−0.011 to 0.019]	0.572	0.002 [−0.009 to 0.013]	0.729	0.003 [−0.007 to 0.013]	0.598
Impulse control	0.035 [−0.009 to 0.080]	0.121	0.009 [−0.028 to 0.047]	0.628	−0.012 [−0.036 to 0.012]	0.336	0.001 [−0.025 to 0.026]	0.952
Substance use	0.011 [−0.008 to 0.029]	0.275	0.002 [−0.013 to 0.018]	0.752	0.002 [−0.013 to 0.017]	0.771	0.004 [−0.009 to 0.016]	0.566

*Notes*: Model 1 only controls for age and gender. Model 2 additionally controls for education, income, job status and living situation. For the discrepancy, both models additionally control for actual SSS. Age of onset and recency are entered into the model as continuous variables.

*p < 0.05, **p < 0.01, ***p < 0.001.

With regard to recency, mental disorders in the year before baseline and in the 1–5 years before baseline were negatively associated with actual SSS for each disorder category (*B* = −1.48 to −0.39, *p* < 0.001–0.049). However, mental disorders that remitted 6 or more years before baseline were no longer significantly associated with actual SSS (*B* = −0.20 to −0.09, *p* = 0.071–0.473), with the exception of impulse control disorders in the 6–10 years before baseline (*B* = −0.73, *p* = 0.025) (Table 4, model 1). Controlling for objective SES generally attenuated the magnitude of associations (Table 4, model 2). There were few associations between recency categories and the actual–desired SSS discrepancy, with only past-year mood or anxiety disorders (*B* = 0.17, *p* = 0.008) and impulse control disorders in the 1–5 years before baseline (*B* = 1.26, *p* = 0.022) being statistically significantly associated with the discrepancy. These associations were unchanged after controlling for objective SES.

Recency was significantly associated with actual SSS among those with any disorder (*B* = 0.015, *p* = 0.005) and marginally significantly so among those with mood or anxiety disorders specifically (*B* = 0.016, *p* = 0.046). After controlling for objective SES, these associations became non-significant (Table 5, model 2). Recency was not significantly associated with the actual–desired SSS discrepancy (*p* = 0.336–0.771, controlling for actual SSS) (Table 5, model 1).

## Discussion

### Principal findings

In this study, we showed that lifetime mental disorders are associated with lower actual SSS and, to a lesser extent, with a slightly larger discrepancy between desired and actual SSS. Thus, people with mental disorders do not come as close to achieving their desired social position as people without mental disorders. Associations for actual SSS were attenuated, but largely persisted after controlling for objective SES, while associations with the actual–desired SSS discrepancy were unchanged after controlling for objective SES. Our study therefore confirms and extends previous work showing that mental health problems are associated with SSS (McLaughlin *et al*., 2012; Honjo *et al*., 2014; Scott *et al*., 2014).

Our analyses using categorical ages of onset showed that participants with mental disorders generally had a lower SSS than healthy participants regardless of age of onset of the disorder. However, our analyses using continuous age of onset within the group of participants with a disorder provided inconclusive evidence that earlier age of onset is associated with lower SSS than later age of onset, given the presence of a disorder. Our categorical analyses also showed that disorders in long-term remission were not associated with significantly lower SSS. The lower SSS experienced by people with recent mental disorders compared to those with long-remitted mental disorders appeared to be at least partly related to lower objective SES, as controlling for objective SES attenuated the association between (continuous) recency and SSS.

To our knowledge, no previous work has examined the discrepancy between *actual* and *desired* SSS. Our finding that people with any lifetime mental disorder have a larger discrepancy than healthy participants suggests that people with lifetime mental disorders are particularly dissatisfied with their position in life, which could potentially contribute to mental health problems. However, associations of mental disorders with the actual–desired SSS discrepancy (after controlling for actual SSS) were generally small in magnitude and only significant for a few specific mental disorders. While participants with mental disorders do have a larger actual–desired SSS discrepancy than healthy participants (results not shown), this is largely explained by differences in actual SSS between participants with and without mental disorders.

Hence, the association between mental disorders and the actual–desired SSS discrepancy may generally be of relatively limited clinical importance compared to the association between mental disorders and actual SSS, although a few specific disorders (conduct disorder and drug dependence) did show quite large associations with the actual–desired SSS discrepancy even after controlling for actual SSS. This contrasts with previous research that found that the discrepancy between actual SSS and a counterfactual SSS (if participants had not become unemployed or had not become single parents) was as strongly associated with depressive symptoms as actual SSS (Euteneuer *et al*., 2019). It is possible that the counterfactual SSS in that study was more salient to participants, given that it represents a plausible alternative reality that was ‘lost’ (upon becoming unemployed or becoming a single parent). The salience of ‘lost’ alternative selves (i.e. clarity of the mental image and frequency of thinking about it) has been related to reduced well-being (King and Smith, 2004; King and Hicks, 2007). The concept of desired SSS used in this study, on the other hand, could be a more nebulous ideal that participants do not have a very clear picture of and that they may or may not have ever realistically expected to achieve.

The finding that disorders in long-term remission were not associated with statistically significantly lower actual SSS is encouraging and suggests that people with mental disorders can fully recover in this regard. This finding concurs with other research showing the desirability of full remission as a treatment outcome to maximise functioning and well-being (Zajecka, 2003). However, we cannot exclude the possibility that the association between recency and SSS is confounded by disorder severity. In general, mild disorders are more likely to remit, while severe disorders are more likely to persist (Spijker *et al*., 2004; Hendriks *et al*., 2013), so the lack of association between long-remitted disorders and actual SSS could also reflect the fact that remitting disorders tend to be milder. Longitudinal research is necessary to disentangle course and severity of the disorder and definitively establish whether the SSS of people with mental disorders tends to normalise after remission.

Although we investigated the association between age of onset and SSS and found a marginally significant positive association with age of onset among those with any mental disorder, the evidence was not sufficiently strong to confidently either rule in or rule out larger associations between early-onset disorders and SSS than between late-onset disorders and SSS. In contrast to previous research (Breslau *et al*., 2008; Lee *et al*., 2009), we found no association between early-onset mental disorders and educational achievement, although early-onset mental disorders were associated with low income. Since education is a plausible mediating variable between early-onset disorders and later SSS, this might explain our inconclusive findings regarding age of onset and SSS. We also found suggestive evidence that the association between age of onset and SSS may be reversed for substance use disorders, with late-onset substance use disorders being more strongly associated with SSS than early-onset disorders. We speculate that this might reflect the fact that early-onset substance use problems are relatively normative and often developmentally limited to adolescence and young adulthood (Maggs and Schulenberg, 2005). However, further research on this topic is necessary.

### Strengths and limitations

One of the strengths of this study is that the NEMESIS-2 cohort is a large sample that is representative of the general population. Furthermore, in contrast to most previous research, we used a validated structured interview (CIDI) to assess diagnosable disorders, rather than using symptom questionnaires. Finally, by examining both actual and desired SSS, we shed some light on whether people with mental disorders adjust their expectations for their life.

A limitation of this study is that we did not investigate the longitudinal and possibly bidirectional relationships between SSS and mental disorders. The observational nature of our study also precludes clear causal inferences. While NEMESIS-2 is a longitudinal cohort, SSS was not assessed at baseline. Consequently, we cannot entirely exclude the possibility that the relationship between baseline mental disorders and follow-up SSS is actually explained by baseline SSS. A limited body of experimental research suggests that an experimental manipulation of SSS resulted in changes in depressive cognitions and stress-reactive ruminations (though no difference in self-reported depressive symptoms) (Schubert *et al*., 2016), implying that lower SSS could have a causal effect on mental health. On the other hand, an experimental manipulation of mood did not result in changes in self-reported SSS (Kraus *et al*., 2013). However, this area of research is still in its infancy, and its relevance to the long-term relationship between SSS and mental health is unclear. Furthermore, we examined a general population cohort and some findings, such as the lack of association between disorders in long-term remission and social status, may not generalise to a more severely affected clinical population. Finally, age of onset and recency were estimated retrospectively. While the CIDI was designed using special probe questions that have been shown to generate more plausible distributions of age of onset (Knäuper *et al*., 1999), some recall bias likely persists.

## Conclusions

In this large, population-representative cohort, we found that lifetime mental disorders were associated with lower SSS and, to a lesser extent, larger discrepancies between actual and desired SSS. The association with actual SSS was somewhat attenuated but persisted after controlling for objective indicators of social status, such as income. Encouragingly, mental disorders that had been in remission for several years were not associated with lower SSS. This suggests that SSS might normalise after remission, and future longitudinal research should investigate this possibility.

## Availability of data and materials

The data on which this manuscript is based are not publicly available. However, data from NEMESIS-2 are available upon request. The Dutch ministry of health financed the data and the agreement is that these data can be used freely under certain restrictions and always under the supervision of the principal investigator (PI) of the study. Thus, some access restrictions do apply to the data.

At any time, researchers can contact the PI of NEMESIS-2 (Margreet ten Have, mhave@trimbos.nl) and submit a research plan, describing its background, research questions, variables to be used in the analyses and an outline of the analyses. If a request for data sharing is approved, a written agreement will be signed stating that the data will only be used for addressing the agreed research questions described and not for other purposes.
